# miR‐30d is related to asbestos exposure and inhibits migration and invasion in NCI‐H2452 cells

**DOI:** 10.1002/2211-5463.12274

**Published:** 2017-08-30

**Authors:** Li Ju, Wei Wu, Xianhong Yin, Yun Xiao, Zhenyu Jia, Jianlin Lou, Min Yu, Shibo Ying, Tianhui Chen, Zhaoqiang Jiang, Wei Li, Junqiang Chen, Xing Zhang, Lijin Zhu

**Affiliations:** ^1^ Institute of Occupational Diseases Zhejiang Academy of Medical Sciences (ZJAMS) Hangzhou Zhejiang China; ^2^ Jiading District Center for Disease Control and Prevention Shanghai China; ^3^ Experimental Animal Center Zhejiang Academy of Medical Sciences (ZJAMS) Hangzhou Zhejiang China

**Keywords:** asbestos, mesothelioma, microRNA‐30d, microRNA, neoplasm invasiveness

## Abstract

Pleural malignant mesothelioma (MM) is a highly aggressive tumor that is typically related to asbestos exposure and has a latency of 20–60 years. Several microRNA contribute to MM initiation and progression, but the mechanisms are not clear. Here, we found that miR‐30d is downregulated in the pleural MM cell line NCI‐H2452, in the plasma of asbestos‐exposed individuals, and in asbestos‐exposed mesothelial cells. Furthermore, we investigated the influence of the overexpression of miR‐30d in pleural MM cells. We demonstrated that miR‐30d overexpression could suppress pleural MM cell proliferation, migration, and invasion *in vitro* and could promote cell apoptosis but could not significantly influence cell cycle. The mRNA and protein expression of vimentin and TWIST1 decreased, and the mRNA expression of CDH1 increased in NCI‐H2452 cells that overexpressed miR‐30d. We therefore conclude that miR‐30d is related to asbestos exposure and inhibits cell migration and invasion by regulating the epithelial–mesenchymal transition in NCI‐H2452 cells.

**Abbreviations**
ATCC: American Type Culture Collection
CDH1: cadherin 1
CT: threshold cycle
EMT: epithelial–mesenchymal transition
miRNA: microRNA
MM: malignant mesothelioma
NC: negative control
NSCLC: non‐small‐cell lung cancer
qPCR: quantitative fluorescence PCR
TWIST1: twist family bHLH transcription factor 1
UICC: Union for International Cancer Control
VIM: vimentin

Pleural malignant mesothelioma (MM), which arises from the cells that line the lung and chest cavity (pleura), is a highly aggressive tumor with a high recurrence rate after surgical resection and is insensitive to chemotherapy and radiotherapy. The median survival time commonly does not exceed 12–18 months after diagnosis 1. Therefore, biomarkers for early detection are imperative even for experienced pathologists. Approximately 80% of pleural MM cases are attributed to asbestos exposure, and the latency after exposure could be 20–60 years 2. Asbestos exposure, genetics, and other factors likely contribute to the etiology of pleural MM 3. Chrysotile, the major type of asbestos, was widely used for commercial purposes during the 20th century, which suggests that the number of mesothelioma cases will increase in the upcoming decades. However, the mechanisms of MM progression remain unclear and few investigations on relative epigenome alterations have been reported.

More recent evidence has indicated that a class of small noncoding RNA referred to as microRNA (miRNA) also contribute to the initiation and progression of a number of tumors, including pleural MM 4. miRNA, a class of 18‐ to 24‐nucleotide (nt)‐long endogenous noncoding RNA, are post‐transcriptional modulators of gene expression. They regulate gene expression by binding to the 3′‐untranslated region (3′UTR) of target genes, resulting in translational repression or mRNA degradation 5. Some miRNA were functionally located in cancer‐associated genomic regions or in fragile sites to act as oncogenes, tumor suppressors, or virulence genes. miRNA disorders are involved in malignant tumor occurrence and development, including pleural MM. Some miRNA were differentially expressed in mesothelial tissue and plasma of patients with MM 6, 7, 8, and might be important indicators for MM diagnosis. Some miRNA were identified as inhibitors in MM migration and invasion *in vitro* 6, 9, which may be helpful in adjunctive therapy for patients with MM. However, more data on the role of dysregulated miRNA that are involved in the carcinogenesis of asbestos exposure with pleural MM are highly warranted.

miR‐30d is a conserved miRNA that belongs to the mir‐30 family, which was first identified by cloning from distinct mouse tissues in 2002 10. miR‐30d may play important roles in several malignant neoplasms, including non‐small‐cell lung cancer (NSCLC) 11, colorectal cancer 12, breast cancer 13, melanoma 14, ovarian carcinoma 15, and prostate cancer 16. To better understand miR‐30d's role in MM, we measured miR‐30d expression in pleural MM cells, chrysotile‐exposed mesothelial cells, and asbestos‐exposed humans. Furthermore, we investigated the growth, metastasis, and epithelial–mesenchymal transition (EMT) marker genes affected by miR‐30d overexpression in NCI‐H2452 cells.

## Materials and methods

### Preparation of chrysotile fibers

In the current study, for chrysotile fibers, we adopted the Union for International Cancer Control (UICC) standard. The chrysotile was a gift from the Society of Fiber Science and Technology, Japan. Chrysotile fibers were weighed, suspended in PBS (Life Technologies/Gibco, Grand Island, NY, USA), and sonicated at 180 W for 30 cycles, with 10 s of ultrasonication and a 5‐s pause using an ultrasonic disrupter (JY92‐IIN; Scientz, Ningbo, China), sterilized by autoclaving, and used at the final concentration as indicated below.

### Examination of asbestos‐exposed subjects

Overall, 117 subjects with asbestos exposure were recruited from residents in the Cixi area, Zhejiang Province, China, where asbestos product manufacturing started in the 1950s. The recruited participants were divided into two groups: healthy subjects without asbestos exposure or asbestos‐related diseases (unexposed group, *n* = 39) and subjects with more than 10 years of asbestos exposure (exposed group, *n* = 78). Asbestos‐exposed subjects were household hand‐spinning women workers and workers in asbestos factories; nonasbestos exposure participants included individuals who were not exposed to asbestos who also had family members who were not exposed to asbestos. There was no significant difference in the distribution of age or gender between the two groups (Table 1). This study was approved by the ethics committee of Zhejiang Academy of Medical Sciences. The written form of informed consent from the aforementioned subjects was obtained during the investigations.

**Table 1:** General characteristics of the study participants

	Unexposed group (*n* = 39)	Exposed group (*N* = 78)	*P* value
Age year (mean ± SD)	68.22 ± 6.55	68.22 ± 6.06	0.97
Gender male/female [*n* (*n*%)]	7/32 (17.95/82.05)	12/66 (15.38/84.62)	0.791
Smoker (*n*)	0	3	0.550
Exposure time (years)		14.37 ± 4.09	

Unexposed group: healthy participants without asbestos exposure; exposed group: subjects exposed to asbestos more than 10 years.

### Cell line and culture

The human pleural MM cell line NCI‐H2452 and normal MeT‐5A pleural mesothelial cells were purchased from American Type Culture Collection (ATCC, Manassas, VA, USA). NCI‐H2452 cells were maintained in RPMI‐1640 medium supplemented with 10% FBS, and MeT‐5A cells were maintained in M199 medium supplemented with 10% FBS. Cells were cultured at 37 °C with 5% CO_2_ and 100% humidity. All cell culture products were purchased from Gibco (Life Technologies/Gibco, Carlsbad, CA, USA).

### Chrysotile treatment to MeT‐5A

MeT‐5A cells were plated at a density of 1 × 10^4^ cells per well into a 96‐well cell culture plate. The next day, cells were exposed to chrysotile at a concentration of 0, 1.25, 2.5, 5, 10, 20, 40, and 80 μg·cm^−2^ (PBS as control) in quintuplicate. After 24 h, cells were tested with the LDH Cytotoxicity Assay Kit (Beyotime, Shanghai, China) according to the manufacturer's instruction. For long‐time exposure, the cells were subcultured twice a week and repeatedly treated with chrysotile (PBS as control) for 24 h every 1 week.

### Transfection with microRNA mimics

In a six‐well cell culture plate, 2 × 10^5^ NCI‐H2452 cells were plated in 2 mL RPMI‐1640 medium with 10% serum. The next day, cells were transfected separately with 50 nm of miR‐30d mimics and nontargeting negative control mimics (NC) by using 3.75 mL of the transfection reagent Lipofectamine 2000 (Life Technologies/Invitrogen, Carlsbad, CA, USA) following the manufacturer's protocol. Transfected cells were harvested at indicated time points to assay cell proliferation, cycle, apoptosis, migration and invasion, mRNA and protein levels.

### RNA‐level detection by real‐time quantitative fluorescence PCR (qPCR)

miRNA was isolated from 200 μL of plasma from 117 human individual blood samples using a mini miRNeasy kit (Qiagen, Hilden, Germany) according to the manufacturer's instructions. Small RNA was extracted according to the manufacturer's protocol. The extracted small RNA was eluted in 14 μL of RNase‐free water. The total RNA of cultured cells was extracted using RNAiso Plus (Takara, Kusatsu, Shiga, Japan).

Reverse transcription of 500 ng miRNA extracted from plasma (5 μL) or total RNA isolated from cells to cDNAs was performed with an miScript^®^ II RT Kit (Qiagen), and cDNA was used as a template to amplify miR‐30d. Quantitative fluorescence PCR (qPCR) was performed with an miScript^®^ SYBR^®^ Green PCR Kit (Qiagen) on an ABI 7500 fast Instrument (Applied Biosystems, Foster, CA, USA) according to the manufacturer's instruction. Triplicate was set up for each sample, and each experiment was repeated at least twice.

Reverse transcription of 500 ng total RNA isolated from NCI‐H2452 cells transfected by miR‐30d mimics was performed with a PrimeScript™ RT reagent Kit (Takara) to cDNAs, and cDNAs were used as templates to amplify cadherin 1 (*CDH1*), vimentin (*VIM*), and twist family bHLH transcription factor 1 (*TWIST1*). PCR primer sequences for *CDH1* were as follows: forward 5′‐TGAAGGTGACAGAGCCTCTGGAT‐3′ and reverse 5′‐TGGGTGAATTCGGGCTTGTT‐3′. PCR primer sequences for *VIM* were forward 5′‐CCAAACTTTTCCTCCCTGAACC‐3′ and reverse 5′‐GTGATGCTGAGAAGTTTCGTTGA‐3′. PCR primer sequences for *TWIST1* were forward 5′‐TGTCCGCGTCCCACTAGC‐3′ and reverse 5′‐TGTCCATTTTCTCCTTCTCTGGA‐3′. The three mRNA were amplified by qPCR with SYBR Premix Ex Taq™ (Takara). Triplicate was set up for each sample, and each experiment was repeated at least twice.

The threshold cycle (CT) is defined as the fractional cycle number at which the fluorescence passes the fixed threshold. The miR‐30d expression levels were normalized to U6 RNA, and the mRNA expression levels were normalized to *ACTIN* mRNA. The relative expression was calculated using the comparative formula 2^−∆∆Ct^. Normalized gene expression levels were quantified to the respective controls.

### Cell proliferation measurement by MTS

NCI‐H2452 cells were seeded in 96‐well cell culture plates with 1 × 10^4^ cells per well in 100 μL medium with 10% serum overnight and transfected with miR‐30d mimics or nontargeting NC in triplicate. Cell viability was measured via the MTS method using the CellTiter 96® AQueous One Solution Cell Proliferation Assay (Promega, Madison, WI, USA) according to the manufacturer's instructions. The absorbance at 490 nm with a 690‐nm reference was measured with a Molecular Device Spectra Max M4 Microplate Reader (Tecan, Morrisville, NC, USA). Relative cell viability was calculated with the NC‐transfected cells as controls. Experiments were repeated at least twice.

### *In vitro* cell migration and invasion assay by transwell and scratch assays

For cell migration by transwell assay, 48 h after transfection, 2 × 10^4^ transfected cells were placed into the upper chamber of an insert (8 μm pore size; BD, Franklin Lakes, NJ, USA) in 200 μL of serum‐free media for 24 h. For the invasion assay, 1 × 10^5^ transfected cells were placed into the upper chamber of an insert coated with Matrigel (BD) in serum‐free media in 200 μL. A total of 500 μL of media containing 10% FBS was added to the lower chamber for 48 h. The cells that remained on the upper membrane were removed with a cotton swab, and the cells that migrated or invaded through the membrane pores were stained with 0.1% crystal violet in methanol, visualized, and counted under an inverted phase contrast microscope.

For the cell migration by scratch assay, 1 × 10^6^ cells were seeded in six‐well plates, cultured overnight, and transfected with miR‐30d mimics or a nontarget NC. When cells reached appropriate confluence, the cell layer was scratched with a sterile plastic tip and washed with culture medium twice and cultured for up to 48 h in serum‐free medium. Images were captured at different time points, 0, 6, 24, 30, and 48 h, via microscopy, to assess the size of the scratch area. Every experiment was repeated at least twice.

### Cell cycle and apoptosis analysis by flow cytometry

Transfected NCI‐H2452 cells were collected by centrifugation, and then, the cell cycle and apoptosis were analyzed as described previously 17. Cell cycle profiles were analyzed by flow cytometry after transfection with miR‐30d mimics and NC for 48 h. Cell apoptosis was detected by flow cytometry coupled with the FITC‐Annexin V Apoptosis Detection Kit (BD Biosciences Pharmingen, San Diego, CA, USA) after transfection with miR‐30d mimics and NC for 72 h following the manufacturer's instructions. Experiments were repeated at least three times.

### Protein‐level detection by western blot analysis and Immunofluorescence Microscopy

Equal amounts of proteins were loaded and separated by 10% SDS/PAGE and then transferred to poly(vinylidene difluoride) membranes in transfer buffer (25 mm Tris, 200 mm glycine, 20% methanol v/v). The membranes were blocked with 5% BSA in TBST (Tris 20 mm, NaCl 137 mm, Tween‐20 0.1%, pH 7.6) for 1 h at room temperature. After washing with TBST, the membranes were incubated in primary antibody at 4 °C overnight followed by incubation with the secondary antibody for 1 h at room temperature. Primary antibodies against CDH1 (BD, diluted 1 : 1000), VIM (BD, diluted 1 : 1000), TWIST1 (Sigma, St. Louis, MO, USA, diluted 1 : 1000), and secondary antibodies (Multisciences, Hangzhou, Zhejiang, China, diluted 1 : 5000) were used. The protein hybrids were scanned with a FluorChem FC2 Imaging System (Alpha, San Antonio, TX, USA). ACTIN (Santa Cruz, Dallas, TX, USA, diluted 1 : 3000) was employed as an internal control for CDH1 and VIM, and histone H3 (Bioworld, Visali, CA, USA, diluted 1 : 1000) was used as an internal control for TWIST1.

Immunofluorescence microscopy was conducted essentially as described previously 18 with slight modification; antibodies against TWIST1 (sigma, 1 : 100) and VIM (BD, 1 : 100) were applied, followed by immunofluorescent staining with goat anti‐mouse IgG, FITC (Multisciences, 1 : 500). Hoechst33342 (Sigma‐Aldrich) was used for nuclear counterstaining. Cell morphology and intracellular localization of protein were analyzed by fluorescence microscopy (Leica DMI4000B, Germany).

### Statistical analysis

Statistical analysis was performed using spss 17.0 software (Armonk, NY, USA). Basic characteristics and asbestos exposure were examined with bivariate analyses, using the chi‐square test. All data were expressed as the mean ± standard deviation (SD). Differential expression of plasma miRNA using qRT‐PCR was evaluated by paired *t*‐tests. Differential expression of cellular miRNA and mRNA detected by qRT‐PCR among the asbestos‐exposed groups and the control was calculated by one‐way ANOVA. Differential expression of RNA and proteins in transfected cells and in the control cells was calculated by unpaired *t*‐tests. A two‐sided *P* < 0.05 was considered statistically significant.

## Results

### miR‐30d was downregulated in the pleural malignant mesothelioma cell line

**Figure 1:**
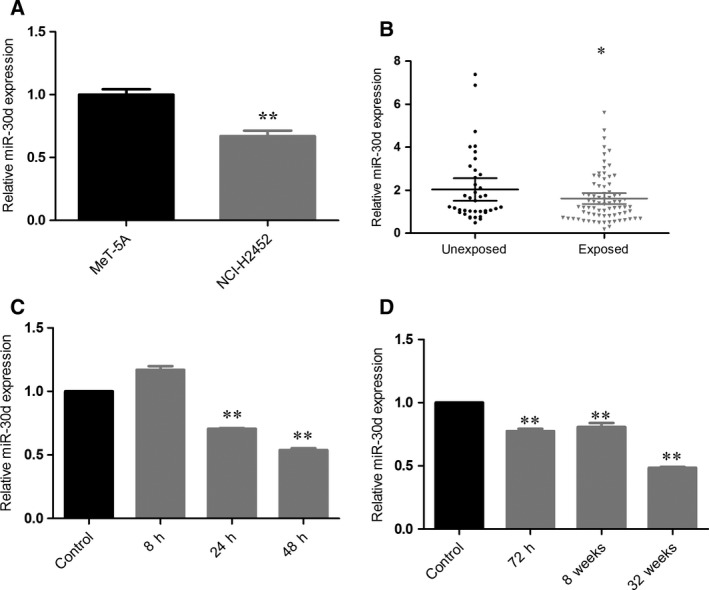
miR‐30d is downregulated in MM cells, chrysotile‐exposed mesothelial cells, and asbestos‐exposed human population. (A) miR‐30d expression in pleural MM cell line NCI‐H2452 and normal human mesothelial cell line MeT‐5A. (B) miR‐30d in plasma of asbestos‐exposed population. (C) Time–response relationship of relative miR‐30d expression in MeT‐5A cells treated with 5 μg·cm^−2^ chrysotile for 8, 24, and 48 h compared with PBS‐treated MeT‐5A control. (D) Time–response relationship of relative miR‐30d expression in MeT‐5A cells treated with 5 μg·cm^−2^ chrysotile for 72 h, 8, and 32 weeks compared with PBS‐treated MeT‐5A control. Expression levels of miR‐30d were determined by qRT‐PCR and normalized against an endogenous control (U6 RNA). Data were represented as the mean ± SD. (*n* = 3). *P < 0.05, **P < 0.01. All assays were performed in duplicate.

We measured miR‐30d expression in the pleural MM cell line NCI‐H2452 and in the normal mesothelial cell line MeT‐5A. The relative expression level of miR‐30d in pleural MM cells was 0.671 compared to MeT‐5A cells (Fig. 1A).

### miR‐30d was downregulated in the plasma of asbestos‐exposed individuals and in chrysotile‐exposed MeT‐5A cells

To explore miR‐30d expression in asbestos‐exposed subjects, the miR‐30d levels in the plasma of 78 asbestos‐exposed individuals and 39 controls were examined. We found that miR‐30d was downregulated in the plasma of asbestos‐exposed subjects. The relative expression level was 0.766 (Fig. 1B).

The time–response relationship of relative miR‐30d expression in MeT‐5A cells treated with 5 μg·cm^−2^ chrysotile for 24, 48, 72 h, 8, and 32 weeks compared with PBS‐treated MeT‐5A control cells for 24, 48, 72 h, 8, and 32 weeks is shown in Fig. 1C,D. The relative expression levels of miR‐30d in chrysotile‐treated cells were 0.705 and 0.538 at 24 and 48 h, respectively, and 0.775, 0.808, and 0.484 at 72 h, 8, and 32 weeks, respectively, compared with MeT‐5A cells. All assays were performed in duplicate.

### Time–response cytotoxicity of chrysotile‐treated MeT‐5A cells

**Figure 2:**
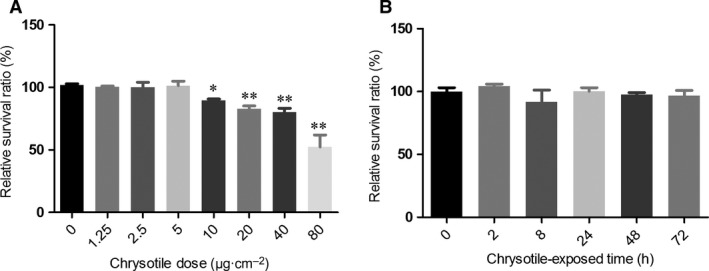
Cell toxicity to MeT‐5A cells treated with chrysotile. (A) Relative survival ratio was determined by LDH assay compared with MeT‐5A control. Chrysotile treatment for 24 h reduced MeT‐5A cell viability in a concentration‐dependent manner with a significant reduction in viability following 10 μg·cm^−2^ chrysotile treatment. (B) Relative survival ratio of MeT‐5A cells treated by 5 μg·cm^−2^ chrysotile was determined by LDH assay compared with MeT‐5A control. There was no significant effect on MeT‐5A viability after treatment by 5 μg·cm^−2^ chrysotile for 24, 48, and 72 h, respectively. *P < 0.05, **P < 0.01.

The cytotoxicity of chrysotile was analyzed with the LDH release assay. The results showed that 24‐h chrysotile treatment reduced MeT‐5A cell line viability in a concentration‐dependent manner. Significant reduction in viability was observed for 10 μg·cm^−2^ chrysotile treatment (Fig. 2A). In addition, there was no significant effect of 5 μg·cm^−2^ chrysotile treatment on MeT‐5A viability after 24, 48, or 72 h (Fig. 2B).

### miR‐30d inhibited cell proliferation, migration, and invasion

**Figure 3:**
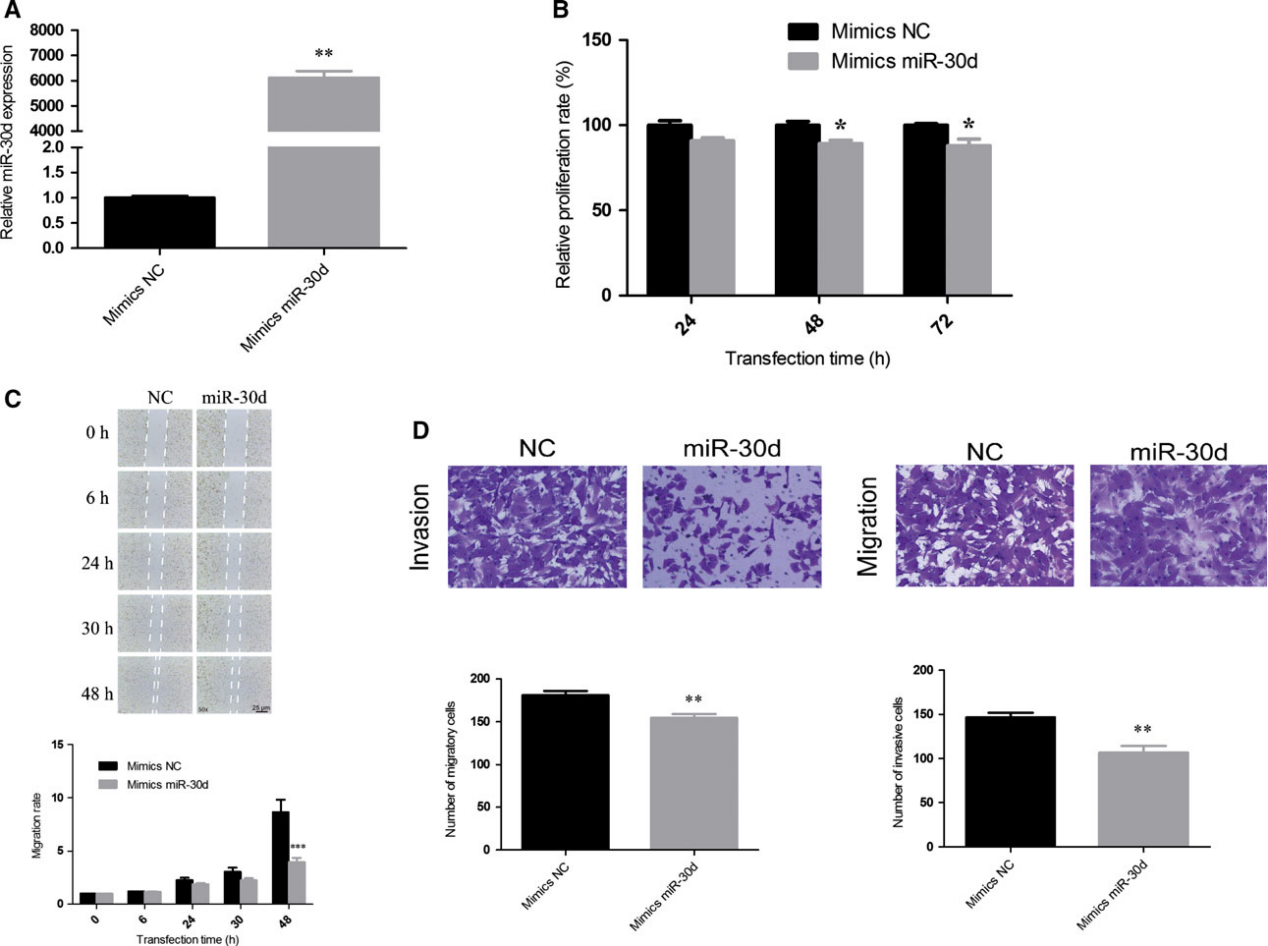
miR‐30d inhibits proliferation, migration, and invasion in NCI‐H2452 cells. (A) The level of miR‐30d in NCI‐H2452 cells was significantly upregulated after transfection with miR‐30d mimics compared with the NC. (B) The proliferation was suppressed in miR‐30d mimics‐transfected NCI‐H2452 cells. Cell proliferation was determined using MTS assays. (C) The wound healing rate in cells transfected with miR‐30d for 48 h significantly decreased. (D) The number of migrating or invading cells in the miR‐30d mimics group significantly decreased compared with the NC. Data were represented as the mean ± SD. *P < 0.05, **P < 0.01, ***P < 0.001. All assays were performed in duplicate.

To better understand the mechanistic role of miR‐30d in mesothelial progression, the pleural MM cell line NCI‐H2452 was transfected with either miR‐30d mimics or a nontargeting mimic control. The overexpression of miR‐30d was confirmed by qPCR as shown in Fig. 3A. To determine the role of miR‐30d in the proliferation of NCI‐H2452 cells, the MTS assay was performed. The relative miR‐30d‐transfected cell proliferation rate was 90.97%, 89.37%, and 88.06% at 24, 48, and 72 h post‐transfection, respectively (Fig. 3B). Furthermore, miR‐30d overexpression suppressed NCI‐H2452 migration and invasion. In the scratch assay, cells in the miR‐30d mimics‐transfected group migrated more slowly than cells in the mimics‐ NC group, which showed that high miR‐30d expression significantly suppressed NCI‐H2452 migration (Fig. 3C). In the transwell invasion and migration assays, upregulated miR‐30d expression inhibited migration and invasion compared with the mimic control group (Fig. 3D; the results of Fig. 3 have been presented in the IASLC 17th World Conference on Lung Cancer, and published online in http://www.jto.org/).

### miR‐30d promoted apoptosis in NCI‐H2452 cells

**Figure 4:**
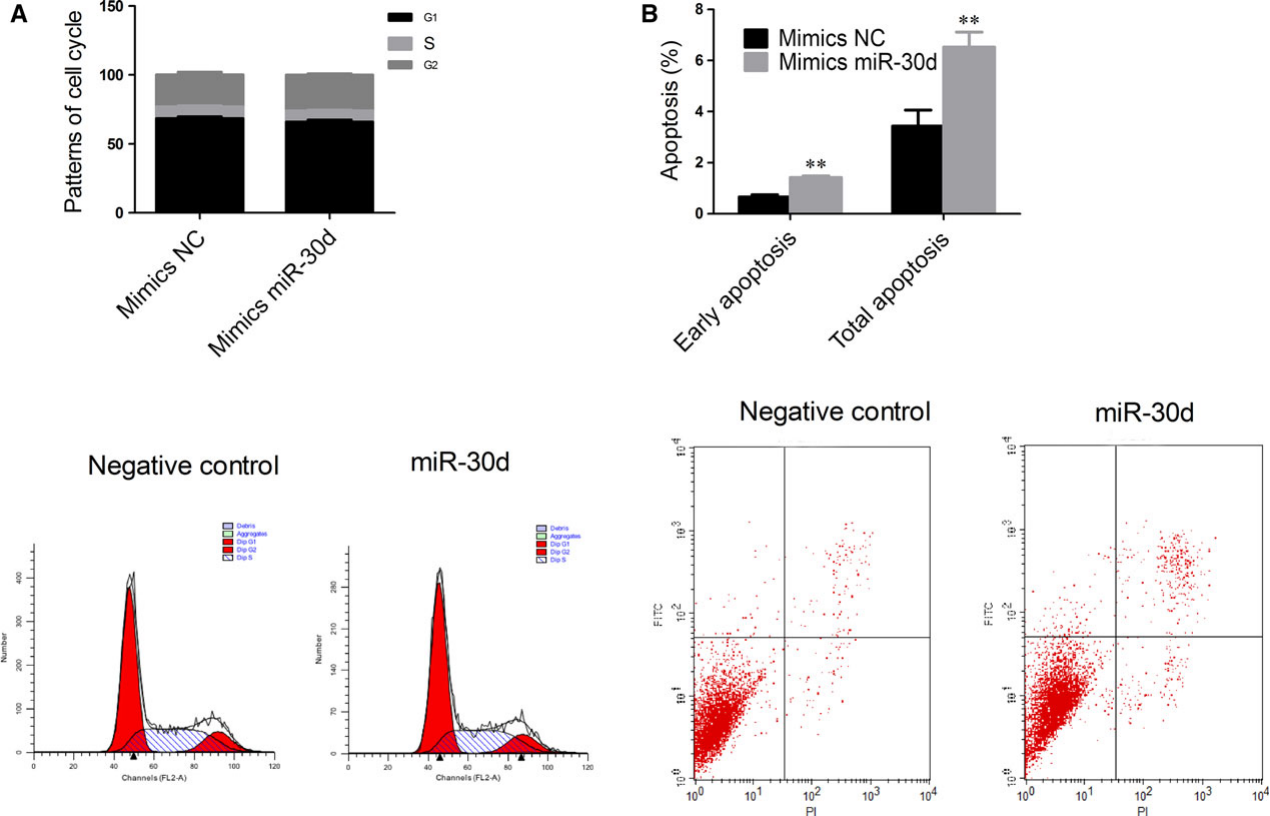
miR‐30d induces apoptosis in NCI‐H2452 cells. (A) miR‐30d did not significantly arrest cell cycle in miR‐30d‐transfected NCI‐H2452 cells compared with the NC. (B) The percentage of early apoptotic cells and total apoptotic cells increased in miR‐30d‐transfected NCI‐H2452 cells compared with the NC. Data were represented as the mean ± SD. **P < 0.01. All assays were performed in duplicate.

To explore the possible mechanism underlying the inhibitory effect of miR‐30d overexpression on cell growth, cell cycle, and apoptosis, analyses were performed. After the upregulation of miR‐30d, there was no significant difference in the percentage of NCI‐H2452 cells that were in G1/S/G2 (Fig. 4A). The results of the apoptotic assay showed that the percentage of cells undergoing early apoptosis and the total apoptotic rate increased (Fig. 4B).

### miR‐30d reverses the expression of the EMT marker gene

**Figure 5:**
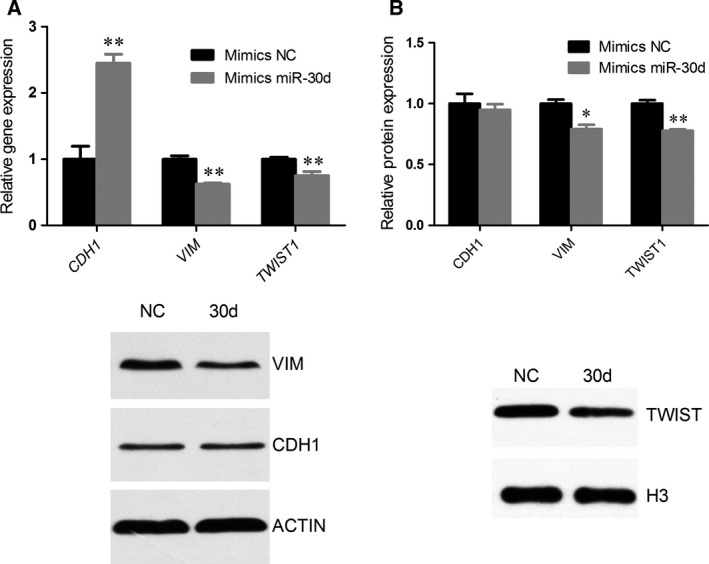
miR‐30d reverses EMT marker gene and protein expression. The level of EMT marker gene in NCI‐H2452 cells was significantly reversed post‐transfection with miR‐30d mimics compared with the NC. (A) Expression levels of CDH1, VIM, and TWIST1 gene were determined by qRT‐PCR and normalized against the endogenous control ACTIN. (B) Expression levels of CDH1, VIM, and TWIST1 proteins were determined by western blot analysis and normalized against the endogenous control ACTIN/H3. (C) The expression of proteins hybridizing bands was detected by western blot analysis. Data were represented as the mean ± SD. *P < 0.05, **P < 0.01. All assays were performed in duplicate.

**Figure 6:**
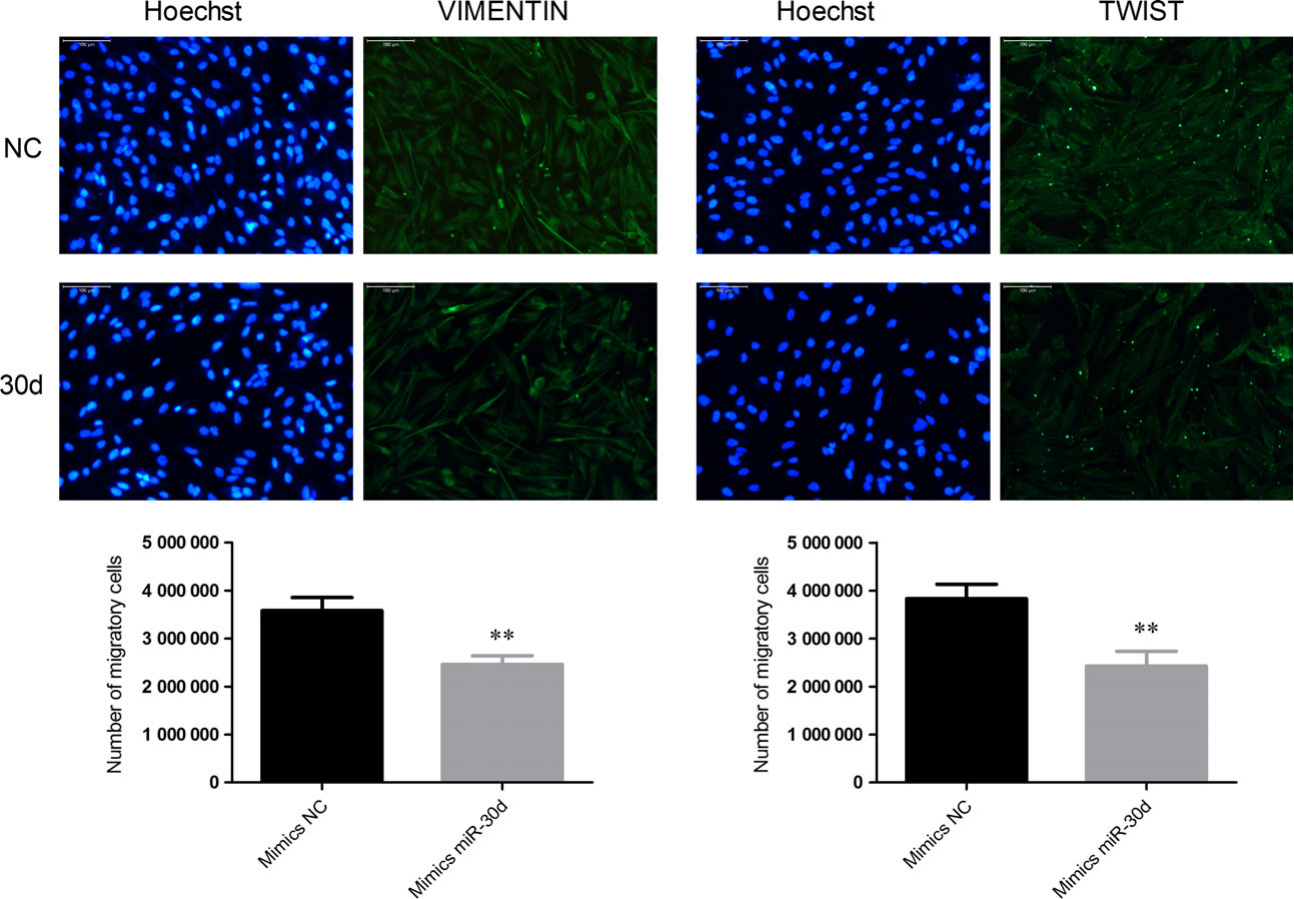
Immunofluorescence microscopy of VIM and TWIST1% mimics miR‐30d led to low expression of VIM and TWIST1 in NCI‐H2452 cells. VIM expression was mainly detected in the cytoplasm, while TWIST1 was detected in the cytoplasm and nuclei in NCI‐H2452 cells. **P < 0.01.

To explore the possible mechanism underlying the inhibitory effect of miR‐30d overexpression on cell migration and invasion, gene and protein expression levels of EMT markers CDH1, VIM, and TWIST1 were examined. The results showed that the gene expression of *CDH1* was upregulated, while *VIM* and *TWIST1* were downregulated, which was quite contrary to the occurrence of EMT; the protein expression of CDH1 was not significantly downregulated, but the protein expressions of VIM and TWIST1 were downregulated (Fig 5). Immunofluorescent staining results showed that mimics miR‐30d led to a low expression of VIM and TWIST1 (Fig. 6) in NCI‐H2452 cells. VIM expression was found in the cytoplasm of NCI‐H2452, while TWIST1 was expressed in the cytoplasm and nuclei.

## Discussion

To the best of our knowledge, this is the first study to determine that miR‐30d was downregulated in pleural MM cells and asbestos‐treated mesothelial cells, and in the plasma of asbestos‐exposed subjects. We demonstrated that miR‐30d overexpression could suppress pleural MM cell proliferation, migration, and invasion *in vitro* and promote cell apoptosis and reverse EMT marker gene expression, suggesting that miR‐30d might be defined as a tumor suppressor miRNA and that its downregulation might contribute to pleural MM occurrence and progression and asbestos carcinogenesis. The versatile functions of miR‐30d in tumor cell proliferation, migration, and invasion suggest its potential use as a prognostic predictor and cancer therapeutic target.

Although a few studies have described dysregulated miRNA in MM, the biological functions of dysregulated miRNA in pleural MM are not well understood. miRNA have potential use for cancer screening, patient surveillance, and diagnosis, and for predicting the response to chemotherapy, and they could also serve as therapeutic targets or molecular biomarkers. With respect to pleural MM, limited data on dysregulated miRNA have been reported over the last few years, as recently reviewed 19. In this study, we found that miR‐30d was downregulated in pleural MM cells, in line with previous studies that reported decreased miR‐31 expression in mesothelioma cells 20, overexpressed 193‐3p in pleural MM 21, and dysregulated miR‐17‐5p, miR‐21, miR‐29a, miR‐30c, miR‐30e‐5p, miR‐106a, and miR‐143 in different histopathological subtypes. With regard to the miR‐30 family 22, miR‐30c, miR‐30e 23, and miR‐30b*^24^ were dysregulated in MM. Therefore, the miR‐30 miRNA family may play an important role in MM biology.

The abnormal expression of miRNA has been reported in many types of cancers, and considerable attention is focused on understanding the role that miRNA play in the process of cancer development 25, 26 rather than the effect of environmental exposure, especially to carcinogens such as asbestos 27. The cumulative experimental data indicate that similar effects are caused by a variety of environmental carcinogens, including polycyclic aromatic hydrocarbons, nitropyrenes, endocrine disruptors, airborne mixtures, carcinogens in food and water, and carcinogenic drugs 28, 29. Consequently, the alteration of miRNA expression is a general event that plays an important pathogenic role to connect exposure to environmental toxic agents with their pathological consequences, mainly including cancer development 28. Pleural MM is highly associated with occupational and/or environmental exposure to asbestos fibers; therefore, asbestos‐exposed mesothelial cells are very good models for the exploration of the mechanism of asbestos carcinogenesis and pleural MM occurrence. In this manuscript, we further showed that miR‐30d was remarkably decreased not only in mesothelioma cells but also in asbestos‐exposed mesothelial cells and asbestos‐exposed humans.

To ensure general consistency between the exposed group and nonexposed group, we chose residents from the same geographical area in the study of plasma from asbestos‐exposed individuals. However, the nonexposed subjects may have been exposed to asbestos in the environment, which could cause asbestos‐related diseases. When asbestos or other naturally occurring asbestos (NOA) is present throughout the United States, Europe, and many other parts of the world, all groups are exposed 30. We could not eliminate the influence of environmental exposure. Furthermore, the fold change was 0.766, and there was some overlap between the two groups. Therefore, the number of subjects and the significance of the results are limited in our study, and more subjects are needed in the future to validate the data. The result was consistent with the results of asbestos‐exposed MeT‐5A cells. The downregulated expression of miR‐30d in MeT‐5A cells induced by acute and chronical asbestos exposure and in pleural MM cells indicated that miR‐30d was involved in asbestos exposure and might be associated with asbestos carcinogenesis. Hence, it might be a biomarker for high risks from asbestos exposure and pleural MM.

miR‐30d has an impact on the dysregulation of such processes as cell proliferation, apoptosis, migration, and tumor growth in lung cancer 31, 32. miR‐30d is involved in cell survival and apoptosis in malignant peripheral nerve sheath tumor cells and in renal cell carcinoma 33, 34. By means of a series of *in vitro* assays, we uncovered that the overexpression of miR‐30d could inhibit cell proliferation, migration, and invasion and promote apoptosis in pleural MM cells. miR‐30d could induce G1/S cell cycle arrest in NSCLC 31 but did not influence cell cycle in pleural MM cells in our study. Uniformly, when KEGG pathways were integrated with miR‐30d's target gene predicted by TargetScan (http://www.targetscan.org/) and Miranda (http://www.microrna.org/), no cell cycle‐related genes were found to be involved (Table S1). Taken together, miR‐30d significantly reduced cell proliferation, migration, and invasion *in vitro*, which indicated that miR‐30d might have a tumor suppressor function.

The target genes of miR‐30d are involved in metabolism, malignant transformation, endocytosis, cell adhesion, the MAPK signaling pathway, the WNT signaling pathway, and the TGF‐beta signaling pathway. miR‐30d downregulates many cancer‐related genes. miR‐30d inhibits renal carcinoma cell proliferation via the regulation of cyclin E2 expression at the post‐transcriptional level 35. miR‐30d post‐transcriptionally suppresses the expression of the oncoprotein metadherin (MTDH) by destabilizing its mRNA in renal cell carcinoma 34. miRNA‐30d induces insulin transcription factor MafA expression and insulin production by targeting mitogen‐activated protein 4 kinase 4 (MAP4K4) in pancreatic beta‐cells 36. miR‐30d inhibits the proliferation and colony formation of anaplastic thyroid carcinoma cells by targeting EZH2 37. We found a downregulation of *VIM* and *TWIST1* genes and proteins and an upregulation of *CDH1* gene when miR‐30d mimics were transfected into the pleural MM cells. CDH1 is an epithelial‐specific junction protein, VIM is a mesenchymal marker, and TWIST1 is an important EMT transcription repressor. The downregulated *CDH1* gene expression and upregulated *VIM* and *TWIST1* gene expression indicated that EMT, which is a process by which epithelial cells lose their cell polarity and cell–cell adhesion, gains migratory and invasive properties, which leads to the acquisition of mesenchymal stem cell characteristics. The differential mRNA and protein expression of CDH1 may be due to regulation in the translation process. miR‐30d reversed EMT gene expression, probably by targeting EMT‐related genes such as *VIM,* which has been identified as a target gene of miR‐30d by the target predicted databases, TargetScan (Fig. S1) and Miranda (Fig. S2), or by targeting some other genes that indirectly influence EMT progress.

## Conclusions

In summary, we found that miR‐30d was downregulated in pleural MM cells, in asbestos‐treated mesothelial cells, and in the plasma of asbestos‐exposed individuals. miR‐30d overexpression could suppress pleural MM cell proliferation, migration, and invasion *in vitro*, promote cell apoptosis, and reverse EMT marker gene expression. miR‐30d could be a tumor suppressor miRNA, and its downregulation might contribute to pleural MM occurrence and progression and asbestos carcinogenesis. Future investigation shall focus on miR‐30d expression in other types of MM cells and pleural MM patients, as well as on its target genes.

## Author contributions

LZ involved in the conception of the study; LJ, WW, and XY performed the experiments and wrote the manuscript; LZ, YX, ZJ, MY, z, and ZJ performed the data analyses; WL contributed reagents/materials/analysis tools; JL, JC, and TC helped perform the analysis with constructive discussions.

## Supporting information


**Fig. S1**. Predicted miRNA targets of miRNA‐30‐5p by TargetscanHuman 7.1.

**Fig. S2**. Predicted miRNA targets relationship of miR‐30d and VIM by Miranda.

**Table S1**. KEGG pathways integrated with miR‐30d's target gene predicted by TargetScan and Miranda.
