# Supplementary figures and images for: miR‐30d is related to asbestos exposure and inhibits migration and invasion in NCI‐H2452 cells
Source: FEBS Open Bio. 2017 Aug 30;7(10):1469–79. doi: 10.1002/2211-5463.12274 (PMC5623706; doi:10.1002/2211-5463.12274)

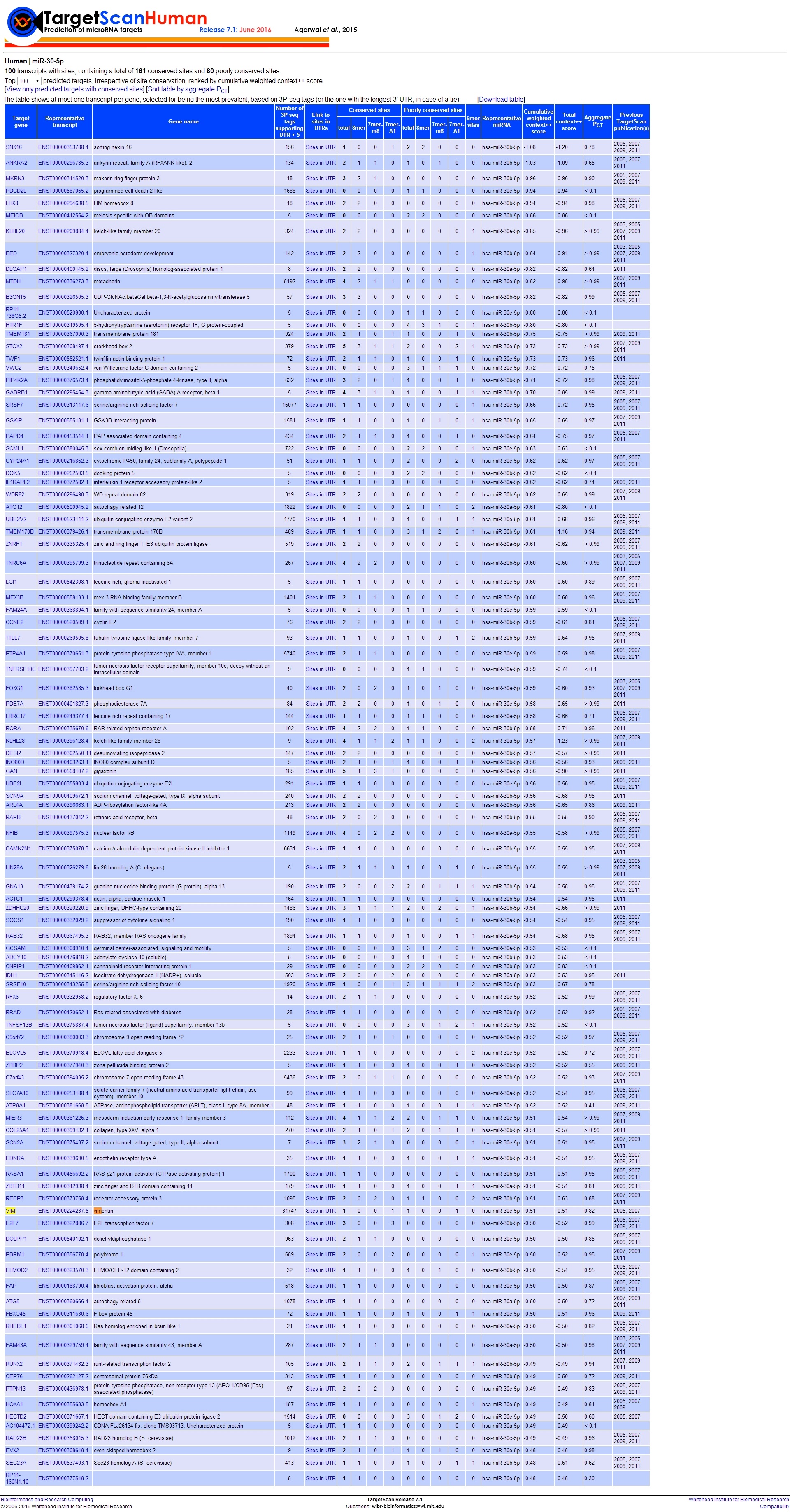

Supplement: Supplementary file 1 — Fig. S1. Predicted miRNA targets of miRNA‐30‐5p by TargetscanHuman 7.1. [file FEB4-7-1469-s001.jpg]

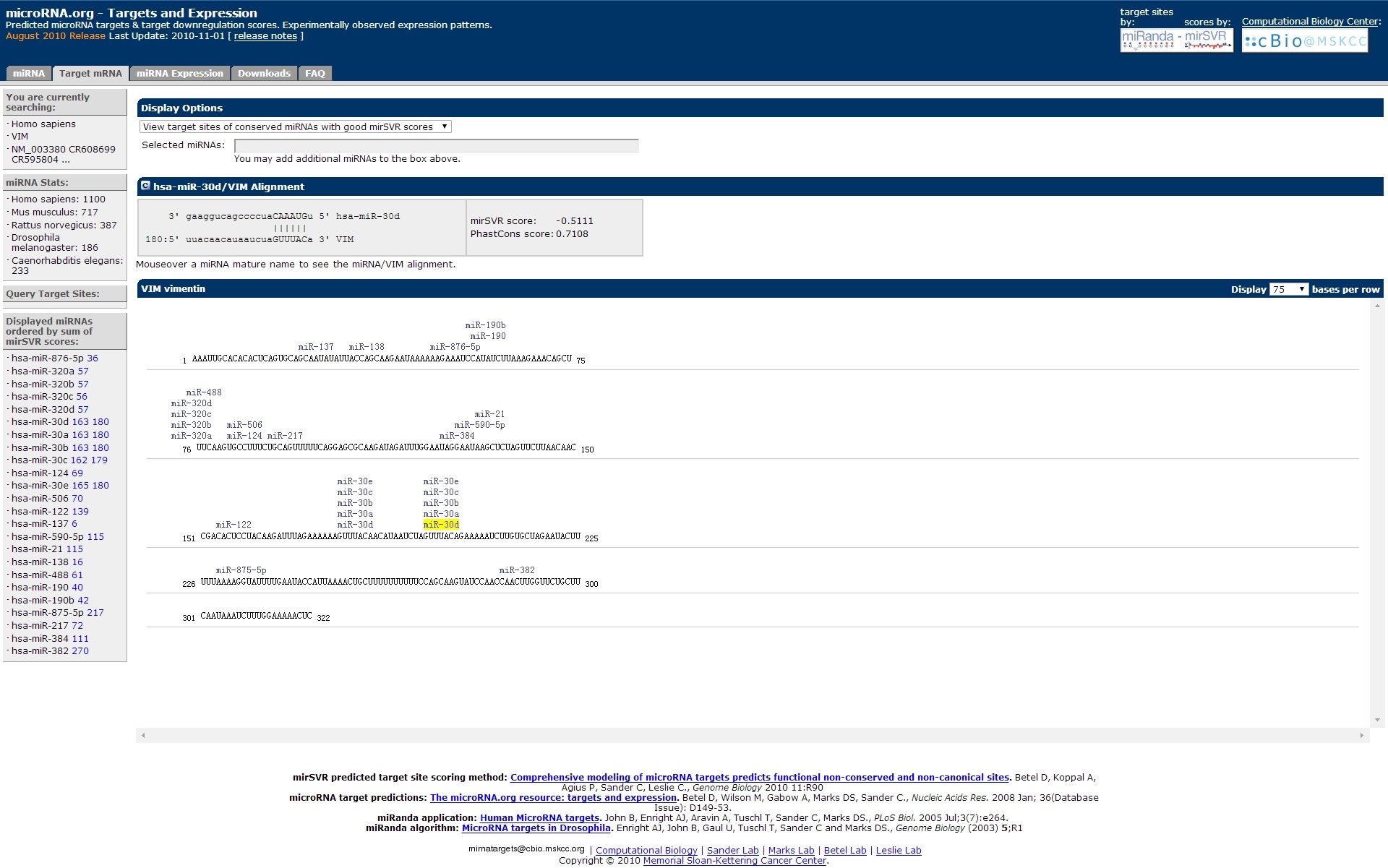

Supplement: Supplementary file 2 — Fig. S2. Predicted miRNA targets relationship of miR‐30d and VIM by Miranda. [file FEB4-7-1469-s002.jpg]
